# Dietary intake is associated with respiratory health outcomes and DNA methylation in children with asthma

**DOI:** 10.1186/s13223-017-0187-8

**Published:** 2017-02-27

**Authors:** L. Montrose, T. J. Ward, E. O. Semmens, Y. H. Cho, B. Brown, C. W. Noonan

**Affiliations:** 1 School of Public Health, University of Michigan, 1420 Washington Heights, Ann Arbor, MI 48109 USA; 2 Center for Environmental Health Sciences, University of Montana, 32 Campus Drive-159 Skaggs, Missoula, MT 59812 USA; 3 Department of Health and Human Performance, University of Montana, 32 Campus Drive, Missoula, MT 59812 USA

**Keywords:** Asthma, Methylation, Spirometry, Diet, Nutrition, Children, Epigenetics, Quality of life

## Abstract

**Background:**

Asthma is an increasingly common chronic disease among children, and data point toward a complex mechanism involving genetic, environmental and epigenetic factors. Epigenetic modifications such as DNA hypo- or hyper-methylation have been shown to occur in response to environmental exposures including dietary nutrients.

**Methods:**

Within the context of the asthma randomized trial of indoor wood smoke (ARTIS) study, we investigated relationships between diet, asthma health measures, and DNA methylation. Asthma health measures included a quality of life instrument, diurnal peak flow variability (dPFV) and forced expiratory volume in the first second (FEV_1_). Dietary intake was assessed with a food frequency questionnaire. Methylation levels of LINE-1 repetitive element and two promoter CpG sites for interferon gamma (IFNγ, -186 and -54) from buccal cell DNA were measured using pyrosequencing assays.

**Results:**

Data were collected on 32 children with asthma living in western Montana who were recruited to the ARTIS study. Selenium and several methyl donor dietary nutrients were positively associated with the asthma quality of life measure. Intake of methyl donating nutrients including folate was positively associated LINE-1 methylation and negatively associated with IFNγ CpG-186. Higher levels of LINE-1 methylation were associated with greater dPFV.

**Conclusion:**

We identified several nutrients that were associated with improved quality of life measures among children with asthma. The IFNγ promoter CpG site -186 but not -54 was associated with the intake of selected dietary nutrients. However, in this small population of children with asthma, the IFNγ promoter CpG sites were not associated with respiratory health measures so it remains unclear through which epigenetic mechanism these nutrients are impacting the quality of life measure. These findings add to the evidence that dietary nutrients, particularly foods containing methyl donors, may be important for epigenetic regulation as it pertains to the control of asthma.

*Trial registration* ClincialTrials.gov NCT00807183. Registered 10 December 2008

**Electronic supplementary material:**

The online version of this article (doi:10.1186/s13223-017-0187-8) contains supplementary material, which is available to authorized users.

## Background

Asthma is an environmentally triggered disease that affects nearly 26 million people in the United States [1]. Dietary intake represents a modifiable environmental exposure that could partially explain the current burden of chronic disease, including asthma, in industrialized countries [2]. Epidemiological studies suggest that dietary patterns are linked to the risk of developing asthma, however, the evidence from longitudinal birth cohorts has not clearly defined the importance of specific nutrients or fully elucidated the mechanistic pathways linking diet to chronic respiratory disease. Further, there have been few studies aimed at determining if nutrient intake contributes to asthma control in children. One potential mechanism whereby dietary intake affects respiratory health in children is through epigenetic modulation of immunoregulatory cytokines.

Significant observational data suggests that dietary status and intake of particular nutrients can affect respiratory health outcomes. Several recent studies have suggested that some dietary nutrients may be protective for respiratory health [3–12]. A recent review concluded that dietary intake in utero and throughout the lifecourse can influence respiratory health status, however definitively assessing causal relationships in human studies is a major challenge [13]. A cross-sectional study by Berthon et al. showed that among asthmatics, a high fat diet was associated with increased airway eosinophilic inflammation, and low fiber intake was associated with poor lung function [14]. Supplementation of dietary folic acid has been successful in the prevention of neural tube defects in the United States. However, longitudinal cohort studies have produced mixed results regarding maternal folic acid supplementation and asthma development [15, 16]. Antioxidants like selenium may play a role in respiratory health through systemic reduction of oxidative stress [17]. In a mouse model of allergic airway disease, a combinatory therapeutic that included selenium attenuated the physiologic airway damage that is typical of this model [18].

The rapidly evolving field of epigenetics has emerged as an appealing potential mechanistic bridge that could link environmental exposures to the development of asthma or the exacerbation of asthma-related symptoms [19]. The exact toxicoepigenetic mechanisms are far from elucidated, but landmark studies using the agouti mouse model have provided solid evidence that environmental exposures can affect phenotype through alterations in DNA methylation patterns [20]. Understanding how and when these mechanisms can impact asthma pathogenesis is paramount. In a mouse model of allergic airway disease, in utero dietary intake of methyl donating nutrients was associated with an enhanced disease phenotype as well as aberrant hypermethylation of runt-related transcription factor 3 (Runx3), a gene known to suppress allergic airway disease [21]. Although the perinatal exposure window may be especially important, data also suggest that environmental exposures could impact health via epigenetic mechanisms throughout the lifecourse. In humans, the production of regulatory T cells (Tregs), which are known to suppress immune responses, is controlled by transcription factor forkhead box p3 (FOXP3) [22]. Nadeau et al. demonstrated that patients with asthma in a polluted environment had a hypermethylated FOXP3 locus profile which was associated with impaired Treg function relative to patients with asthma in a less polluted area [23].

The relationship between dietary intake and epigenetic modifications is complex and compounded by sensitivity to timing of exposure (e.g. prenatal, postnatal, adolescent, or adult). Nevertheless, human and mouse data indicate several dietary nutrients play a role in epigenetic mechanisms [24], thus it is possible that nutrient intake is related to asthma pathogenesis through the epigenetic regulation of key genes. Asthma is phenotypically characterized by a shift toward type 2 T helper (Th2) polarization and consequently type 1 T helper (Th1) cell cytokines such as interferon gamma (IFNγ) play a critical role as counter regulators in the allergic asthma pathway [25, 26]. For example, in a follow-up study of adults recruited as children with a history of wheeze, those with persistent asthma were compared to those with resolved asthma to characterize the Th1/Th2 response following exposure to house dust mite allergen [27]. Smart et al. found that those with persistent asthma had much weaker Th1 responses and concluded that a measured decrease in IFNγ production in this group could be a major factor underpinning the presence of severe and chronic asthma symptoms. Meng et al. investigated the effect of diet on IFNγ production in humans and showed that cells extracted and purified from non-asthmatic adults produced differential amounts of IFNγ [28]. Interestingly, Meng found that the amounts of IFNγ were associated with intake of specific dietary variables and predicted upper respiratory tract infection incidence. Finally, a series of studies using either a ragweed or dust mite-sensitized mouse model of asthma showed that pre-treatment with a DNA adjuvant known to result in Th1 biased immune status with marked overproduction of IFNγ resulted in an ameliorated lung inflammatory phenotype [29, 30]. Thus IFNγ is a relevant candidate gene that plausibly exists in the mechanistic pathway linking dietary intake to respiratory health via epigenetic regulation of the Th1/Th2 cytokine balance.

Poor asthma control is associated with school absences, higher health care costs and worse long-term health outcomes. An understanding of the association between a child’s recent dietary history and respiratory health measures could lead to important intervention strategies to improve outcomes among children with asthma. In this study we aimed to evaluate the relationship between a priori selected nutrients and asthma health. Although the link between current dietary status and asthma health is not clear, evidence suggests a potential role for an epigenetic mechanism. In addition to a measure of global gene methylation, IFNγ was chosen as a candidate gene because of its well-established role in the Th1/Th2 balance.

## Methods

### Study overview

Participants were recruited from the asthma randomized trial of indoor wood smoke (ARTIS) study. The rationale and methods for the ARTIS study have been described previously [31–33]. The ARTIS study included 114 children with asthma (ages 6–17) from 97 homes in Montana, Idaho, and Alaska. This parent study was designed to test an indoor air quality intervention, and homes were assigned to either a placebo arm or an air filter intervention. Two in-home data collection visits occurred in each of two consecutive winter periods with the intervention occurring between these winter periods. The subcohort recruited to participate in this diet and epigenetics study included 32 participants living in western Montana who had been recruited in the final 2 years of the 5-year ARTIS study. Additional file 1: Figure S1 indicates when spirometry measurements, buccal cells, and food frequency questionnaires (FFQ) were administered. For the purpose of the currently described study, only data that was collected in conjunction with a FFQ was considered. In Additional file 1: Figure S1, this would be visits B and D. Health outcomes measures included a quality of life instrument and self-monitoring of spirometry measures using a peak flow meter. Buccal cell samples were collected for evaluating epigenetic markers. Anthropometric measures determined by trained staff using a digital scale and stadiometer along with the participant’s gender and date of birth were used to calculate body mass index (BMI) percentile using the U.S. Centers for Disease Control and Prevention (CDC) calculator [34]. The study was approved by the University of Montana Institutional Review Board. In addition to the informed consent procedures for the parent study, children were separately assented to participate in this diet and epigenetics study and parents signed a parental permission and informed consent form.

### Dietary nutrient collection

Dietary data was collected using the 2004 Block Kids FFQ (NutritionQuest, Berkeley, CA, USA) to characterize dietary intake among participants. This instrument has been validated in children, ages 6–17 years old [35–38]. The questionnaire includes 77 food items. In addition to intake of standard nutrients, this instrument was used to estimate intake of micronutrients that participate in the one-carbon metabolism pathway (i.e. betaine, choline, folate, etc.). These nutrients are important for the generation of methyl groups and are therefore potentially relevant to DNA methylation markers. The questionnaire was administered to each participant by trained staff using serving size visual aides, and parents were asked to assist their child with portion size recognition and remembering foods they ate during the last week. Questionnaires were processed by NutritionQuest, and the resulting data were analyzed at the University of Montana.

### Health outcome measures collected in parent study

The pediatric asthma quality of life questionnaire (PAQLQ) is a 23-item asthma-specific battery which provides domain scores for symptoms (10 items), activity limitation (5 items), and emotional function (8 items) [39]. The total PAQLQ score and each domain score are calculated as mean scores ranging from one to seven with seven as the optimal score. The PAQLQ has been validated as an evaluative tool to measure within participant changes over time due to treatment, and changes in this scale of 0.5 or more points are clinically significant [39].

Using the PiKo-1 m (Ferraris Respiratory, Ayer, MA, USA) participants performed a test twice daily, in the morning and in the evening, for a period of 2 weeks. These 2-week periods were initiated at the beginning of each air sampling event. For each test, the child’s parent records the observation as it appears on the meter and these observations are later checked for accuracy against the digital log of the instrument. The instrument records the best result for both peak expiratory flow (PEF) and forced expiratory volume in one second (FEV_1_). Outcomes from these measures include average morning PEF and FEV_1_, average evening PEF and FEV_1_, and diurnal PEF variability (dPFV).

### Cell collection, DNA extraction, and pyrosequencing

Buccal cells were collected from the participant’s cheek by trained staff using a cytology brush and stored in Cell Lysis Solution (Qiagen, Valencia, CA, USA) at room temperature until all samples were collected. In compliance with this protocol, all samples were processed within 24 months from the day of collection. DNA from the buccal cells was extracted using Gentra Puregene Buccal Cell DNA Kit (Qiagen, Valencia, CA, USA) according to the manufacturer’s instructions. The quantity of the purified DNA was measured using a Nanodrop spectrophotometer (Thermo Scientific, Wilmington, DE) and then stored at −20 °C. DNA bisulfite treatment was carried out using the EZ DNA Methylation-Direct Kit (Zymo Research, Irvine, CA) according to the manufacturer’s instruction, and stored at −20 °C. Pyrosequencing assay was used to measure methylation levels of LINE-1 repetitive element and the promoter region of INFγ. Briefly, 50 µg of bisulfite-modified DNA was PCR amplified by polymerase chain reaction (PCR) using specific primers (Additional file 1: Table S1) and the PyroMark PCR kit (Qiagen, Valencia, CA, USA). After annealing, pyrosequencing was conducted using a Pyromark Q96 MD (Qiagen, Valencia, CA, USA). Samples were run in duplicate and only samples with a coefficient of variation less than 5% were used in the final analysis. Epitect (Qiagen, Valencia, CA, USA) bisulfite treated controls, which include a methylated and unmethylated human genome sample, along with a no template control were used on each plate.

### Statistical analysis

All analyses were conducted using SAS v9.4 (Cary, NC, USA). To evaluate if cross sectional measurements of IFN CpG sites are correlated with each other and/or correlated with LINE1 global methylation we estimated Pearson correlation coefficients using the first available observation for each participant (n = 32). A subset of 17 macro- and micronutrients from the total 73 nutrient variables generated by the FFQ were chosen after a literature review of diet as it relates to asthma. Relationships between a priori selected dietary nutrients and both epigenetic markers and asthma outcomes were considered in separate models using all available and complete data, which included multiple visits for some participants. Their associations with continuous epigenetic markers (i.e. global and gene-specific methylation) and asthma measures were evaluated using generalized estimating equations (GEE), which account for correlations between repeated measures on the same participant. Tertiles of dietary nutrients were included in analyses as three-level indicator variables to investigate potentially non-linear relationships with epigenetic and asthma outcomes. Analyses were adjusted for age (continuous) and gender. Although this diet and epigenetics repeated measures study was not directly related to the indoor air quality intervention study, we included in our models indicators for pre- versus post-intervention winter and home intervention assignment (i.e., placebo versus air filter). Inclusion of the following potential confounders: presence of cat or dog in home (yes or no), family income (above or below $50,000) and parent education (college degree or no college degree) appreciably impacted parameter estimates. Therefore, the final model included age, gender, winter and intervention group assignment, presence of cat or dog, income and education. We investigated relationships between epigenetic markers and asthma measures in a similar manner. Due to the number of comparisons (n = 264), a false discovery rate correction [40] was applied and adjusted p-values (q-values) were calculated for each relationship where the GEE model was used. A threshold for significance was set at q < 0.20 which means we accept that 20% of the observed significant relationships (i.e. 3.4 out of 17) could be false positives.

## Results

A subset of 32 children from the ARTIS cohort participated in this study of diet, asthma health and epigenetics and was included in the analyses described here. Diet data was collected once per winter in conjunction with buccal cells, PAQLQ and spirometry and therefore, only these ‘complete’ visits were considered in the analysis. Approximately 63% of subcohort, or 20 participants, had both a year one and year two ‘complete’ visit, while 12 participants only had one ‘complete’ visit, which occurred in either year one or year two, for a total of 52 observations. Reasons for these 12 participants having one rather than 2 years of data included missing data, participant not available during scheduled visit, or the participant chose to only participate in one year of the study. Moreover, in the final GEE models, which were adjusted for several covariates, one participant (two observations) was excluded because income and education data was missing, therefore the results from these models include 50 observations from 31 participants. Ages ranged from 8 to 17 years and 47% were male (Table 1). The study population was 94% non-Hispanic white. The mean (sd) BMI percentile was 70.6 (20.1) and 34% (n = 11) were above the 85th percentile, which is considered overweight according to the CDC [34]. Baseline asthma-related respiratory health values can be found in Table 1. Mean values for both dPFV and FEV_1_ were at the approximate threshold used to designate poor asthma control [41]. Mean (sd) LINE-1 methylation was 65.3% (3.4) with a range of 56.1–73.2%. Mean (sd) IFNγ CpG-54 was 79.6% (4.5) with a range of 68.6–92.4%. Mean (sd) IFNγ CpG-186 was 70.1% (6.6) with a range of 49.1–81.6%. IFNγ CpG-54 and IFNγ CpG-186 observations were moderately correlated with each other (r = 0.42; p = 0.02) as were IFNγ CpG-54 and LINE-1 methylation (r = 0.44; p = 0.01). IFNγ CpG-186 and LINE-1 methylation were not significantly correlated (r = 0.26; p = 0.15).

**Table 1:** Selected characteristics of subset of ARTIS participants included in epigenetic study

	N	Mean	SD	Range
Age	32	12.8	2.5	8.0–17.0
Gender				
Male	15 (46%)			
Female	17 (53%)			
Ethnicity				
Non-Hispanic	30 (94%)			
BMI percentile	32	70.6	24.1	5.8–99.0
dPFV	32	20.0	14.0	2.0–66.0
% Predicted morning FEV_1_	32	81.7	19.7	26.9–112.9
% Predicted evening FEV_1_	32	82.3	19.3	19.3–110.1
PAQLQ	32	5.6	1.1	3.1–7.0
LINE-1 (%)	32	65.3	3.4	56.1–73.2
IFNγ-54 (%)	32	79.6	4.5	68.6–92.4
IFNγ-186 (%)	32	70.1	6.6	49.1–81.6

*SD* standard deviation, *BMI* body mass index, *dPFV* evening to morning peak flow variability, *FEV*_*1*_ forced expiratory volume in 1 s, *PAQLQ* pediatric asthma quality of life questionnaire, *IFNγ* interferon gamma

### Evaluating dietary nutrients with respect to respiratory health

When considered across categories of calculated intake, most dietary nutrients failed to show a consistent association with respiratory health measures, but several differences in PAQLQ scores were observed between participants in the highest third versus the lowest third of intake for some nutrients (Table 2). Phosphatidylcholine was the only selected nutrient that was associated with any of the three pulmonary function measure assessed. Children in the middle tertile relative to the lowest had 16.04% point (95% CI 3.31, 28.78; q = 0.16) higher % predicted evening FEV_1_. Intake of selenium and folate was associated with better PAQLQ scores. Specifically, participants with the highest tertile of selenium and folate intake had 1.4 unit (95% CI 0.90, 1.91; q = 0. 01) and 0.92 unit (95% CI 0.31, 1.53; q = 0.11) higher PAQLQ scores, respectively. Additionally, nutrients in the one-carbon metabolism cycle, phosphocholine (1.11 unit higher PAQLQ score; 95% CI 0.23, 1.98; q = 0.16) and betaine (0.98 unit higher PAQLQ score; 95% CI 0.30, 1.66; q = 0. 13) were positively associated with PAQLQ.

**Table 2:** The relationship between select nutrients and asthma health measures by dietary tertiles in ARTIS where the lowest tertile of intake (T1) is the reference group

Asthma health measure	dPFV		% Predicted morning FEV_1_		% Predicted evening FEV_1_		PAQLQ	
Dietary factor	Estimate (95% CI)	q-value	Estimate (95% CI)	q-value	Estimate (95% CI)	q-value	Estimate (95% CI)	q-value
Kilocalories								
T3:T1	−3.18 (−11.41, 5.06)	0.80	13.09 (−4.04, 30.22)	0.61	4.18 (−14.08, 22.44)	0.87	0.62 (−0.25, 1.48)	0.68
T2:T1	−0.50 (−8.78, 7.78)	0.98	0.83 (−13.94, 15.60)	0.98	7.04 (−8.31, 22.40)	0.76	−0.34 (−1.20, 0.51)	0.79
Protein								
T3:T1	−6.96 (−20.40, 6.48)	0.72	19.26 (0.48, 38.05)	0.33	10.96 (−2.58, 24.51)	0.55	0.70 (−0.16, 1.56)	0.55
T2:T1	−3.42 (−14.56, 7.73)	0.83	6.31 (−8.30, 20.92)	0.77	1.14 (−12.42, 14.70)	0.97	0.59 (−0.09, 1.27)	0.51
M-fats								
T3:T1	−2.87 (−10.62, 4.87)	0.81	11.64 (−6.82, 30.11)	0.71	8.55 (−10.72, 27.81)	0.77	0.39 (−0.31, 1.09)	0.71
T2:T1	−0.57 (−8.81, 7.67)	0.98	3.89 (−9.58, 17.36)	0.83	3.26 (−9.25, 15.77)	0.85	0.52 (−0.03, 1.07)	0.41
S-fats								
T3:T1	−3.99 (−15.87, 7.89)	0.83	16.85 (1.36, 32.35)	0.29	11.73 (0.65, 22.82)	0.33	−0.16 (−0.82, 0.50)	0.87
T2:T1	−6.56 (−16.72, 3.59)	0.70	16.67 (2.96, 30.37)	0.28	10.19 (−0.41, 20.80)	0.41	−0.05 (−0.77, 0.67)	0.98
Omega 3:6 ratio								
T3:T1	−1.25 (−10.54, 8.03)	0.92	2.42 (−11.59, 16.44)	0.90	−0.39 (−13.84, 13.07)	1.00	−0.13 (−1.18, 0.93)	0.92
T2:T1	−1.29 (−12.09, 9.51)	0.92	5.40 (−4.00, 14.80)	0.71	1.46 (−6.18, 9.10)	0.90	0.11 (−0.54, 0.75)	0.90
Selenium								
T3:T1	5.44 (−2.98, 13.86)	0.70	6.63 (−6.31, 19.56)	0.73	6.37 (−6.57, 19.30)	0.74	1.40 (0.90, 1.91)	0.01*
T2:T1	2.52 (−4.56, 9.59)	0.82	0.20 (−14.11, 14.51)	1.00	2.91 (−12.96, 18.77)	0.90	−0.56 (−1.59, 0.47)	0.71
Fiber								
T3:T1	−0.39 (−9.02, 8.25)	0.99	7.75 (−5.27, 20.78)	0.71	5.86 (−8.02, 19.74)	0.77	0.66 (−0.52, 1.84)	0.71
T2:T1	0.63 (−9.85, 11.11)	0.98	−5.36 (−17.98, 7.27)	0.77	−3.60 (−15.79, 8.59)	0.83	0.32 (−0.38, 1.02)	0.76
Folate								
T3:T1	−3.43 (−14.76, 7.89)	0.83	10.07 (−7.95, 28.09)	0.71	3.75 (−10.53, 18.02)	0.85	0.92 (0.31, 1.53)	0.11*
T2:T1	−3.03 (−14.23, 8.17)	0.85	−0.03 (−17.50, 17.44)	1.00	−0.24 (−13.99, 13.50)	1.00	−0.25 (−0.83, 0.35)	0.78
Methionine								
T3:T1	−4.83 (−13.90, 4.24)	0.72	14.81 (−0.25, 29.88)	0.36	11.07 (−4.02, 26.17)	0.65	0.52 (−0.37, 1.41)	0.71
T2:T1	1.87 (−7.53, 11.28)	0.90	5.22 (−12.12, 22.57)	0.83	7.29 (−10.16, 24.73)	0.77	−0.06 (−1.58, 1.46)	0.99
Free choline								
T3:T1	2.44 (−4.68, 9.56)	0.83	11.20 (−6.45, 28.86)	0.70	7.69 (−10.98, 26.36)	0.78	0.00 (−1.34, 1.34)	1.00
T2:T1	5.35 (−3.47, 14.17)	0.71	−2.64 (14.74, 9.47)	0.88	−0.21 (−13.39, 12.97)	1.00	−0.31 (−1.26, 0.64)	0.83
Glycpp-choline								
T3:T1	2.84 (−5.75, 11.42)	0.83	−3.05 (−18.17, 12.07)	0.89	−3.64 (−19.88, 12.61)	0.88	0.61 (−0.17, 1.39)	0.59
T2:T1	−1.81 (−9.22, 5.59)	0.87	2.88 (−9.04, 14.80)	0.87	3.70 (−8.10, 15.50)	0.83	0.12 (−0.41, 0.65)	0.87
Pp-choline								
T3:T1	4.54 (−3.07, 12.15)	0.71	−2.95 (−12.99, 7.08)	0.83	−5.21 (−15.57, 5.14)	0.73	1.11 (0.23, 1.98)	0.16*
T2:T1	−1.03 (−7.55, 5.50)	0.90	−10.81 (−27.27, 5.64)	0.69	−12.35 (−29.79, 5.09)	0.68	0.50 (−0.61, 1.60)	0.77
Ppt-choline								
T3:T1	0.36 (−6.86, 7.58)	0.98	11.60 (−1.45, 24.65)	0.48	−1.13 (−19.21, 16.94)	0.98	0.24 (−0.86, 1.35)	0.88
T2:T1	5.71 (−2.92, 14.33)	0.69	−3.17 (−15.51, 9.17)	0.85	16.04 (3.31, 28.78)	0.16*	−0.19 (−0.58, 0.21)	0.76
Total choline								
T3:T1	1.33 (−6.50, 9.16)	0.90	8.61 (−7.92, 25.14)	0.72	5.96 (−12.23, 24.15)	0.83	0.18 (−1.26, 1.61)	0.92
T2:T1	3.76 (−4.42, 11.94)	0.76	−6.68 (−20.25, 6.90)	0.74	−2.06 (−16.74, 12.62)	0.92	−0.38 (−1.35, 0.59)	0.80
Betaine								
T3:T1	−2.62 (−9.35, 4.10)	0.80	12.85 (0.98, 24.72)	0.29	9.13 (−1.59, 19.85)	0.51	0.98 (0.30, 1.66)	0.13*
T2:T1	4.20 (−6.18, 14.58)	0.79	−9.34 (−21.55, 2.86)	0.61	−9.10 (−19.78, 3.59)	0.68	0.21 (−0.56, 0.98)	0.85
Vitamin B-2								
T3:T1	−0.95 (−8.43, 6.54)	0.92	−4.74 (−18.10, 8.61)	0.82	3.81 (−10.97, 18.58)	0.85	0.40 (−0.66, 1.46)	0.80
T2:T1	1.66 (−8.13, 11.44)	0.90	8.98 (−7.57, 25.52)	0.71	−4.04 (−17.65, 9.56)	0.83	−0.12 (1.12, 0.88)	0.92
Vitamin B-6								
T3:T1	2.53 (−5.37, 10.43)	0.83	4.19 (−10.21, 18.59)	0.83	0.26 (−13.43, 13.94)	1.00	0.46 (−0.21, 1.13)	0.69
T2:T1	4.64 (−3.10, 12.38)	0.71	−4.96 (−20.50, 10.57)	0.83	0.81 (−14.85, 16.46)	0.98	−0.65 (−1.20, 0.00)	0.36
Vitamin B-12								
T3:T1	−0.02 (−8.26, 8.22)	1.00	6.42 (−8.93, 21.77)	0.77	1.70 (−11.74, 15.13)	0.92	0.79 (0.09, 1.50)	0.29
T2:T1	1.53 (−7.17, 10.23)	0.90	−2.40 (−16.46, 11.66)	0.90	−3.38 (−16.85, 10.10)	0.86	0.29 (−0.53, 1.11)	0.82

*M-fats* monosaturated fats, *S-fats* saturated fats, *Glycpp-choline* glycerophosphocholine, *Pp-choline* phosphocholine, *Ppt-choline* phosphotidylcholine, *dPFV* evening to morning peak flow variability, *FEV*_*1*_ forced expiratory volume in 1 s, *PAQLQ* pediatric asthma quality of life questionnaire

* q-value < 0.20

### Evaluating dietary nutrients with respect to methylation outcomes

Intake of several nutrients was associated with LINE-1 methylation and methylation at CpG promoter site IFNγ-186, but not for IFNγ-54 (Table 3). Children in the highest tertile of kilocalories (3.2% points higher methylation; 95% CI 0.82, 5.58; q = 0.16) or the middle tertile of protein (2.67% points higher methylation; 95% CI 0.62, 4.71; q = 0.16) had higher LINE-1 methylation. Similarly, those in the highest tertile of methyl donating nutrients free choline (2.18% points higher methylation; 95% CI 0.54, 3.82; q = 0.16), total choline (2.60% points higher methylation; 95% CI 0.60, 4.60; q = 0.16) and folate (4.29% points higher methylation; 95% CI 2.25, 6.34; q = 0.01) also had higher LINE-1 methylation. Intake within the middle tertile of kilocalories (4.56% points lower methylation; 95% CI −7.44, −1.69; q = 0.09) and folate (4.05% points lower methylation; 95% CI −6.18, −1.19; q = 0.02) compared to the lowest was associated with less IFNγ-186 methylation. However, intake within middle tertile of monosaturated fat intake (6.88% points higher methylation; 95% CI 3.11, 10.62; q = 0.02) was associated with more IFNγ-186 methylation. Children in the highest tertile of betaine intake (4.34% points lower methylation; 95% CI −7.25, −1.42; q = 0.12) and both the middle and highest tertile of vitamin B6 intake (6.57% points lower methylation; 95% CI −10.65, −2.48; q = 0.09 and 6.63% points lower methylation; 95% CI −11.13, −2.14; q = 0.12, respectively) had less IFNγ CpG-186 methylation.

**Table 3:** The relationship between select nutrients and DNA methylation markers by dietary tertiles in ARTIS, where the lowest tertile of intake (T1) is the reference group

Epigenetic measure	IFNγ CpG-54		IFNγ CpG-186		LINE-1	
Dietary factor	Estimate (95% CI)	q-value	Estimate (95% CI)	q-value	Estimate (95% CI)	q-value
Kilocalories						
T3:T1	0.46 (−2.46, 3.38)	0.90	−0.11 (−4.35, 4.13)	1.00	3.20 (0.82, 5.58)	0.16*
T2:T1	−0.40 (−2.87, 2.07)	0.90	−4.56 (−7.44, −1.69)	0.09*	0.00 (−2.45, 2.46)	1.00
Protein						
T3:T1	2.60 (−0.90, 6.10)	0.65	−0.04 (−3.41, 3.32)	1.00	1.62 (−0.79, 4.03)	0.69
T2:T1	2.17 (−0.46, 4.80)	0.55	2.02 (−1.24, 5.27)	0.71	2.67 (0.62, 4.71)	0.16*
M-fats						
T3:T1	−1.10 (−3.70, 1.51)	0.77	4.77 (−0.04, 9.57)	0.36	1.14 (−1.23, 3.51)	0.76
T2:T1	−2.42 (−4.74, −0.11)	0.33	6.86 (3.11, 10.62)	0.02*	1.12 (−1.00, 3.24)	0.72
S-fats						
T3:T1	−0.75 (−3.11, 1.61)	0.83	5.11 (−1.08, 11.31)	0.55	1.52 (−1.09, 4.13)	0.71
T2:T1	1.02 (−1.91, 3.96)	0.82	3.00 (−2.48, 8.48)	0.71	1.26 (−1.45, 3.97)	0.76
Omega 3:6 ratio						
T3:T1	1.44 (−1.23, 4.10)	0.71	2.29 (−1.85, 6.44)	0.71	−0.09 (−2.70, 2.52)	0.99
T2:T1	3.72 (0.44, 7.01)	0.29	0.24 (−4.72, 5.20)	0.98	0.96 (−1.03, 2.95)	0.74
Selenium						
T3:T1	0.34 (−2.45, 3.13)	0.92	−1.40 (−5.17, 2.38)	0.81	2.32 (−0.15, 4.79)	0.45
T2:T1	1.40 (−0.60, 3.40)	0.68	−2.38 (−5.01, 0.24)	0.45	−0.18 (−2.69, 2.33)	0.98
Fiber						
T3:T1	0.53 (−2.31, 3.37)	0.90	−4.67 (−9.09, −0.24)	0.33	1.89 (−1.02, 4.80)	0.69
T2:T1	0.19 (−2.29, 2.68)	0.98	−2.52 (−6.54, 1.49)	0.71	−0.27 (−2.70, 2.16)	0.94
Folate						
T3:T1	2.93 (0.06, 5.80)	0.36	2.46 (−2.25, 7.17)	0.72	4.29 (2.25, 6.34)	0.01*
T2:T1	1.95 (−0.03, 3.93)	0.36	−4.05 (−6.18, −1.91)	0.02*	0.46 (−1.24, 2.16)	0.85
Methionine						
T3:T1	0.63 (−2.42, 3.68)	0.89	−2.70 (−6.86, 1.45)	0.69	1.12 (−1.78, 4.03)	0.80
T2:T1	0.43 (−2.25, 3.11)	0.90	−1.38 (−5.68, 2.92)	0.83	1.58 (−1.04, 4.21)	0.71
Free choline						
T3:T1	1.33 (−1.08, 3.74)	0.71	−2.61 (−5.84, 0.63)	0.55	2.18 (0.54, 3.82)	0.16*
T2:T1	1.29 (−1.03, 3.60)	0.71	−3.28 (−6.33, −0.24)	0.29	0.01 (−1.82, 1.85)	1.00
Glycpp-choline						
T3:T1	0.44 (−2.23, 3.12)	0.90	−2.81 (−5.95, 0.33)	0.48	0.66 (−1.48, 2.80)	0.83
T2:T1	1.68 (−0.54, 3.89)	0.65	−0.31 (−3.84, 3.22)	0.97	0.24 (−1.74, 2.21)	0.92
Pp-choline						
T3:T1	0.50 (−2.65, 3.65)	0.90	−1.77 (−5.27, 1.73)	0.73	0.41 (−1.43, 2.25)	0.88
T2:T1	1.47 (−0.52, 3.46)	0.65	−1.54 (−5.83, 2.75)	0.82	−2.08 (−4.41, 0.24)	0.48
Ppt-choline						
T3:T1	1.49 (−1.08, 4.06)	0.71	−2.33 (−6.06, 1.39)	0.71	0.64 (−1.66, 2.94)	0.84
T2:T1	−0.78 (−3.40, 1.84)	0.83	−1.55 (−6.86, 3.76)	0.83	−1.66 (−4.03, 0.71)	0.68
Total choline						
T3:T1	1.29 (−1.06, 3.64)	0.71	−3.47 (−6.68, −0.26)	0.29	2.60 (0.60, 4.60)	0.16*
T2:T1	0.85 (−1.41, 3.11)	0.80	−3.58 (−6.59, −0.58)	0.28	0.42 (−1.88, 2.73)	0.90
Betaine						
T3:T1	−0.00 (−2.50, 2.49)	1.00	−4.34 (−7.25, −1.42)	0.12*	1.18 (−1.25, 3.62)	0.74
T2:T1	1.52 (−1.21, 4.26)	0.71	1.28 (−4.28, 1.73)	0.77	1.65 (−0.61, 3.91)	0.65
Vitamin B-2						
T3:T1	0.62 (−2.44, 3.68)	0.89	−4.19 (−7.91, −0.48)	0.29	2.47 (−0.37, 5.32)	0.51
T2:T1	1.41 (−0.74, 3.56)	0.69	−4.03 (−7.86, −0.20)	0.33	0.23 (−2.48, 2.94)	0.97
Vitamin B-6						
T3:T1	2.05 (−1.04, 5.14)	0.69	−6.63 (−11.13, −2.14)	0.12*	1.44 (−1.65, 4.52)	0.76
T2:T1	0.66 (−1.80, 3.12)	0.85	−6.57 (−10.65, −2.48)	0.09*	0.13 (−2.39, 2.65)	0.98
Vitamin B-12						
T3:T1	1.12 (−2.31, 4.55)	0.83	−2.31 (−7.00, 2.39)	0.74	0.59 (−2.43, 3.60)	0.90
T2:T1	2.21 (−0.48, 4.91)	0.55	1.84 (−2.37, 6.05)	0.77	0.87 (−2.09, 3.82)	0.83

*M-fats* monosaturated fats, *S-fats* saturated fats, *Glycpp-choline* glycerophosphocholine, *Pp-choline* phosphocholine, *Ppt-choline* phosphotidylcholine, *IFNγ* interferon gamma

* q-value <0.20

### Evaluating DNA methylation with respect to respiratory health

We investigated the relationships between methylation markers and asthma-related respiratory health measurements (Table 4). A one-percentage point increase of LINE-1 methylation was associated with a 1.24 percentage point (95% CI 0.31, 2.16; q = 0.16) increase in dPFV. Neither IFNγ CpG-54 nor-186 methylation was associated with the respiratory health measures evaluated in this study.

**Table 4:** The relationship between epigenetic measurements and asthma health outcomes in ARTIS

Epigenetic marker	dPFV		% Predicted morning FEV_1_		% Predicted evening FEV_1_		PAQLQ	
	Estimate (95% CI)	q-value	Estimate (95% CI)	q-value	Estimate (95% CI)	q-value	Estimate (95% CI)	q-value
LINE-1	1.24 (0.31, 2.16)	0.16*	−0.89 (−3.18, 1.41)	0.80	−1.19 (−3.41, 1.03)	0.72	0.04 (−0.11, 0.20)	0.84
IFNγ CpG-186	0.58 (0.06, 1.09)	0.29	−0.53 (−1.79, 0.72)	0.77	−0.57 (−1.62, 0.48)	0.72	0.03 (0.00, 0.07)	0.44
IFNγ CpG-54	0.22 (−0.74, 1.19)	0.87	−0.57 (−1.92, 0.77)	0.77	−0.84 (−2.10, 0.42)	0.69	0.02 (−0.07, 0.11)	0.85

*dPFV* evening to morning peak flow variability, *FEV*_*1*_ forced expiratory volume in 1 s, *PAQLQ* pediatric asthma quality of life questionnaire, *IFNγ* interferon gamma

* q-value <0.20

## Discussion

In this study of dietary nutrients, DNA methylation and asthma-related respiratory health outcomes, we observed positive associations between several nutrients related to one-carbon metabolism (e.g. folate, phosphocholine and betaine) and the PAQLQ score. These nutrients were not similarly associated with better self-monitored spirometry outcomes, suggesting that the nutrients may positively influence asthma quality of life through another mechanism. The composite PAQLQ score is comprised of symptom, activity and emotion domains; however, a post hoc analysis, which substituted individual domains for the composite PAQLQ in a model with dietary nutrients (i.e. those nutrients that had significant relationships with composite PAQLQ), revealed no difference for the impact of dietary intake on individual domains. Furthermore, the individual domains were highly correlated with one another (data not shown) and therefore it is unclear which of these domains may be more affected by diet.

Folate (or folic acid) is one of the most prominently studied methyl donors and is known to play a role in allergic asthma [42]. Many studies have investigated the efficacy of methyl donating nutrient supplementation to reduce the risk of asthma development. To date the results have been inconclusive (see reviews [2, 43]). Many of these supplementation studies have been either limited by ethical concerns or underpowered. We observed that folate intake was associated with a higher PAQLQ score in children with asthma. Based on the most recent reviews, the evidence suggests that early life exposure to folate has no major effects on asthma outcomes later in life [44]. However, relevant to our findings, few studies have investigated the relationship between folate intake and asthma measures in adolescent children with established asthma [45]. Folate deficiency has been associated with asthma-related symptoms and exacerbations [46–48]. Folate and betaine are involved in DNA methylation through the formation of S-adenosylmethionine and homocysteine metabolism and therefore have the potential to affect gene expression, thereby influencing asthma pathogenesis [43]. In addition to methyl donors, selenium was also positively associated with PAQLQ score. Fabian et al. found that children with asthma compared to healthy control children had lower plasma levels of selenium and higher exhaled nitric oxide, a marker of poor lung health [49]. However, a group of Swedish researchers found no impact of selenium intake on allergic disease in young children [50]. The inconclusive results regarding the impact of selenium intake on allergic asthma could be attributed to the fact that while selenium does have antioxidant properties it also has the ability to upregulate some immune responses [17, 51]. In our analysis selenium status is associated with better asthma quality of life measures, however, this nutrient was not associated with LINE-1 or IFNγ methylation profiles.

Among the dietary nutrients investigated in this study only phosphatidylcholine was modestly associated with self-administered spirometry measures, specifically higher evening FEV_1_. Phosphatidylcholine is phospholipid and a major dietary source of choline, which is involved in one-carbon metabolism. Phospholipids can also impact T cell function in a number of ways including membrane fluidity and gene expression, which could have indirect immunomodulatory effects [52]. Therefore, our observation of a positive association between phosphatidylcholine and FEV_1_ in these children could be reflective of reduced lung inflammation. However, this association was not consistent across tertiles of phosphatidylcholine intake nor was there a consistent response across the different FEV_1_ measures, evening versus morning. These concerns together with the studies that have linked phosphatidylcholine to cardiovascular disease [53] and related inflammatory symptoms [54], suggest that the association between phosphatidylcholine intake and FEV_1_ should be interpreted with caution.

In addition to evaluating the relationship between nutritional intake and asthma health, a potential epigenetic mechanism was examined by determining if global methylation or IFNγ promoter methylation was associated with nutritional intake. Global methylation, though informative, is difficult to interpret in the context of respiratory health and may be even more complex in this cohort of children with asthma. Few studies have looked at buccal DNA LINE-1 global methylation in healthy children. A study of 57 healthy girls aged from 6 to 15 investigated LINE-1 global methylation in saliva samples and the average (SD) was 75.2 (3.4) [55]. The cells collected from buccal and saliva should be similar, however the mean LINE-1 methylation in our study overall was considerably lower, which could be attributed to asthma status. It is clear from the literature that intake of methyl donors can result in measureable changes to the mammalian epigenome [20]. Our study shows that dietary intake of folate, free choline, and total choline is positively associated with LINE-1 methylation. By convention, an increase in global methylation is thought to be protective, while a shift toward genome-wide hypomethylation is often associated with a poor health outcome or disease [56, 57]. Nevertheless, our data suggested that global methylation was positively associated with dPFV, an indicator of airway hyper-reactivity. This finding could be a characteristic of the study population, which had relatively low average global methylation. Further, DNA methylation is dynamic and global methylation is a reflection of the epigenetic changes occurring at many gene locations.

Our study specifically focused on IFNγ as a candidate gene, hypothesizing that this gene would lie in the mechanistic pathway linking dietary intake to asthma health outcomes in children. Previous studies have established that IFNγ CpG-54 and -186 (-53 and -190 are the corresponding murine CpGs) are relevant to allergic outcomes in animal models [58, 59] and humans [60–62]. In the mouse, Jones et al. showed that these CpGs are functionally relevant (i.e., methylation status affects transcription of the IFNγ gene) and that de novo methylation of these sites plays a key role in Th2 polarization at least within the CD4+ T cells [63]. In an human asthma cohort, Lovinsky-Desir et al. showed that there are differential methylation profiles for these CpGs relative to age, sex and tissue type [64]. For example, when methylation profiles of buccal cells and CD4+ lymphocytes isolated from whole blood were compared, IFNγ CpG-186 was correlated for males but not females. Further, methylation values for IFNγ CpG-54 and -186 were correlated for children and adults in CD4+ lymphocytes but only for adults in buccal cells. White et al. investigated IFNγ promoter methylation profiles by in vitro polyclonal expansion of CD4+ and CD8+ T cells sorted from peripheral blood mononuclear cells [62]. When samples collected from adolescent children were stratified by atopic status, the authors found that, for CD8+ T cells under Th1 polarizing conditions, IFNγ CpG sites -54 and -186 were less methylated in the non-atopic children.

When evaluating the impact of diet on IFNγ promoter methylation, we found that only IFNγ CpG-186 methylation patterns were affected by selected nutrients. We observed that intake of kilocalories and three methyl donating nutrients was associated with less IFNγ CpG-186 methylation, while children who had higher intake of monosaturated fats had more IFNγ CpG-186 methylation. Based on the functional data available for this CpG site, which we note does not come from buccal cells, we speculate that a negative association between a dietary nutrient and methylation at this site could impact the Th1/Th2 balance by increasing the expression of IFNγ. Overall nutrient intake has previously been linked to IFNγ production [65]. However, overnutrition is not likely a preferable or effective asthma intervention especially due to the potential links between obesity, inflammation, and asthma. Monosaturated fat was the only nutrient we found to be positively associated with IFNγ-186 methylation. In a study of approximately 1200 adolescent children conducted in Taiwan, intake of monosaturated fats was inversely associated with risk of asthma [66]. By contrast, a study of nearly 4000 adult European participants found that intake of monosaturated fats was positively associated with allergic sensitization [67].

IFNγ CpG promoter methylation at site -54 and -186 was not associated with respiratory health measures or PAQLQ. This suggests that the positive relationship revealed between PAQLQ composite score and intake of selenium, folate, phosphocholine, and betaine may not be working directly through epigenetic modification of these specific sites as we had hypothesized.

### Limitations and cautions

We evaluated several dietary macro- and micronutrients in this study, but these factors likely include only a portion of the exogenous factors that could influence DNA methylation in this population. While FFQs are an accepted and validated method for acquiring personal dietary information, we note that the portion sizes and specific foodstuffs were self-reported by the participants with assistance and input from parents. Though it is widely accepted, BMI may actually be a poor indicator for obesity in children and adolescents who have large, lean body mass from physical activity, high muscularity, or frame size. By focusing on select candidate DNA methylation markers, we recognize that numerous inflammatory pathways involving diet and asthma may not have been captured. We also are limited in our interpretation of the DNA methylation data because we did not measure IFNγ expression or protein levels in these samples. For example, we found some dietary factors to be negatively associated with DNA methylation, which could be informative for asthma intervention strategies, but such interpretations require further assessment as methylation changes do not necessarily translate to functional changes in the target tissue. Finally, although we accounted for false discoveries, we recognize that several statistical tests were performed and would expect some significant results due to chance alone. Thus these observations should be considered exploratory and requiring of further study in other populations.

## Conclusions

Within this cohort of childhood asthmatics, we sought to identify dietary nutrients that may be beneficial for respiratory health. In addition we measured LINE-1 and IFNγ (CpG-54 and -186) methylation levels to identify pathways whereby diet influences the health among children with asthma. In this study, selenium and several nutrients involved in the one-carbon metabolism pathway were associated with improved asthma quality of life measures. Furthermore, these data showed that some dietary constituents were associated with both global and gene specific methylation in children with asthma. The two IFNγ CpG sites that were investigated appear to be uniquely affected by intake of micro- and macronutrients.

## Additional file

**Additional file 1: Table S1.** Primers and amplification conditions for PCR and pyrosequencing experiments. **Figure S1.** Description of ARTIS sub-study design.

## Abbreviations

ARTIS: asthma randomized trial of indoor wood smoke
BMI: body mass index
CDC: U.S. Centers for Disease Control and Prevention
CI: confidence interval
CpG: regions of DNA where a cytosine nucleotide is followed by a guanine nucleotide
DNA: deoxyribonucleic acid
dPFV: diurnal peak flow variability
FEV_1_: forced expiratory volume in the first second
FFQ: food frequency questionnaires
FOXp3: transcription factor forkhead box p3
GEE: generalized estimating equations
glycpp-choline: glycerophosphocholine
IFNγ: interferon gamma
LINE-1: long interspersed nuclear elements
M-fats: monosaturated fats
NO_2_: nitrogen dioxide
PAQLQ: pediatric asthma quality of life questionnaire
PCR: polymerase chain reaction
PEF: peak expiratory flow
PM: particulate matter
PM_2.5_: fine PM or particles less than 2.5 micrometers in aerodynamic diameter
Pp-choline: phosphocholine
Ppt-choline: phosphotidylcholine
Runx3: runt-related transcription factor 3
S-fats: saturated fats
SD: standard deviation
Tregs: regulatory T cells
Th1: type 1 T helper
Th2: type 2 T helper
USEPA: U.S. Environmental Protection Agency

## References

[1] Akinbami LJ (2012). Trends in asthma prevalence, health care use, and mortality in the United States, 2001–2010. NCHS Data Brief.

[2] Han YY (2013). Diet and asthma: vitamins and methyl donors. Lancet Respir Med.

[3] Thuesen BH (2010). Atopy, asthma, and lung function in relation to folate and vitamin B(12) in adults. Allergy.

[4] Mehta AK (2010). Choline attenuates immune inflammation and suppresses oxidative stress in patients with asthma. Immunobiology.

[5] Barros R (2008). Adherence to the Mediterranean diet and fresh fruit intake are associated with improved asthma control. Allergy.

[6] Barros R (2011). Dietary intake of alpha-linolenic acid and low ratio of n-6:n-3 PUFA are associated with decreased exhaled NO and improved asthma control. Br J Nutr.

[7] Castro-Rodriguez JA (2008). Mediterranean diet as a protective factor for wheezing in preschool children. J Pediatr..

[8] Chatzi L (2007). Diet, wheeze, and atopy in school children in Menorca, Spain. Pediatr Allergy Immunol.

[9] Chatzi L (2008). Mediterranean diet in pregnancy is protective for wheeze and atopy in childhood. Thorax.

[10] de Batlle J (2008). Mediterranean diet is associated with reduced asthma and rhinitis in Mexican children. Allergy.

[11] Garcia-Marcos L (2007). Relationship of asthma and rhinoconjunctivitis with obesity, exercise and Mediterranean diet in Spanish schoolchildren. Thorax.

[12] Romieu I (2009). Dietary intake, lung function and airway inflammation in Mexico City school children exposed to air pollutants. Respir Res.

[13] Berthon BS, Wood LG (2015). Nutrition and respiratory health-feature review. Nutrients.

[14] Berthon BS (2013). Investigation of the association between dietary intake, disease severity and airway inflammation in asthma. Respirology.

[15] Cortessis VK (2012). Environmental epigenetics: prospects for studying epigenetic mediation of exposure–response relationships. Hum Genet.

[16] Lovinsky-Desir S, Miller RL (2012). Epigenetics, asthma, and allergic diseases: a review of the latest advancements. Curr Allergy Asthma Rep.

[17] Norton RL, Hoffmann PR (2012). Selenium and asthma. Mol Aspects Med.

[18] Bansal P (2014). Intranasal administration of a combination of choline chloride, vitamin C, and selenium attenuates the allergic effect in a mouse model of airway disease. Free Radic Biol Med.

[19] North ML, Ellis AK (2011). The role of epigenetics in the developmental origins of allergic disease. Ann Allergy Asthma Immunol..

[20] Dolinoy DC (2008). The agouti mouse model: an epigenetic biosensor for nutritional and environmental alterations on the fetal epigenome. Nutr Rev.

[21] Hollingsworth JW (2008). In utero supplementation with methyl donors enhances allergic airway disease in mice. J Clin Invest.

[22] Lloyd CM, Hawrylowicz CM (2009). Regulatory T cells in asthma. Immunity.

[23] Nadeau K (2010). Ambient air pollution impairs regulatory T-cell function in asthma. J Allergy Clin Immunol..

[24] Friso S, et al. One-carbon metabolism and epigenetics. Mol Aspects Med. 2016. 10.1016/j.mam.2016.11.007 27876555

[25] Calderon C (2009). T-cell cytokine profiles are altered in childhood asthma exacerbation. Respirology.

[26] Crinnion WJ (2012). Do environmental toxicants contribute to allergy and asthma?. Altern Med Rev.

[27] Smart JM (2002). Polyclonal and allergen-induced cytokine responses in adults with asthma: resolution of asthma is associated with normalization of IFN-gamma responses. J Allergy Clin Immunol.

[28] Meng H (2015). In vitro production of IL-6 and IFN-gamma is influenced by dietary variables and predicts upper respiratory tract infection incidence and severity respectively in young adults. Front Immunol.

[29] Mohammadi-Shahrokhi V, et al. Immunomodulatory effects of adjuvants CPG, MPLA, and BCG on the Derp2-induced acute asthma at early life in an animal model of BALB/c Mice. Inflammation. 2016. 10.1007/s10753-016-0476-2 27896542

[30] Santeliz JV (2002). Amb a 1-linked CpG oligodeoxynucleotides reverse established airway hyperresponsiveness in a murine model of asthma. J Allergy Clin Immunol.

[31] Noonan CW, Ward TJ (2012). Asthma randomized trial of indoor wood smoke (ARTIS): rationale and methods. Contemp Clin Trials.

[32] Ward TJ (2017). Efficacy of interventions targeting household air pollution from residential wood stoves. J Expo Sci Environ Epidemiol.

[33] Semmens EO (2015). Indoor particulate matter in rural, wood stove heated homes. Environ Res.

[34] BMI percentile calculator for child and teen. https://nccd.cdc.gov/dnpabmi/calculator.aspx. Accessed 1 Dec 2016.

[35] Block G (1994). Revision of dietary analysis software for the health habits and history questionnaire. Am J Epidemiol.

[36] Block G, DiSogra C (1995). WIC dietary assessment validation study. Final report.

[37] Block G (1992). Comparison of two dietary questionnaires validated against multiple dietary records collected during a 1-year period. J Am Diet Assoc.

[38] Block G (1990). Validation of a self-administered diet history questionnaire using multiple diet records. J Clin Epidemiol.

[39] Juniper EF (1996). Measuring quality of life in children with asthma. Qual Life Res.

[40] Benjamini Y, Hochberg Y (1995). Controlling the false discovery rate—a practical and powerful approach to multiple testing. J R Stat Soc Series B-Methodol.

[41] Lung National Heart, Institute Blood (2007). Expert panel report 3: guidelines for the diagnosis and management of asthma.

[42] Sharma S, Litonjua A (2014). Asthma, allergy, and responses to methyl donor supplements and nutrients. J Allergy Clin Immunol.

[43] Blatter J (2013). Folate and asthma. Am J Respir Crit Care Med.

[44] Crider KS (2013). Prenatal folic acid and risk of asthma in children: a systematic review and meta-analysis. Am J Clin Nutr.

[45] Lin JH (2013). Relationships between folate and inflammatory features of asthma. J Allergy Clin Immunol.

[46] Matsui EC, Matsui W (2009). Higher serum folate levels are associated with a lower risk of atopy and wheeze. J Allergy Clin Immunol..

[47] Farres MN (2011). Study of folate status among Egyptian asthmatics. Intern Med.

[48] Blatter J (2016). Folate deficiency, atopy, and severe asthma exacerbations in Puerto Rican children. Ann Am Thorac Soc.

[49] Fabian E (2013). Nutritional supplements and plasma antioxidants in childhood asthma. Wien Klin Wochenschr.

[50] Rosenlund H (2012). Antioxidant intake and allergic disease in children. Clin Exp Allergy.

[51] Hoffmann PR (2008). Selenium and asthma: a complex relationship. Allergy.

[52] Pollock AH (2016). Prolonged Intake of dietary lipids alters membrane structure and T cell responses in LDLr−/− Mice. J Immunol.

[53] Wang Z (2011). Gut flora metabolism of phosphatidylcholine promotes cardiovascular disease. Nature.

[54] de Pablo MA, Puertollano MA, Alvarez de Cienfuegos G (2002). Biological and clinical significance of lipids as modulators of immune system functions. Clin Diagn Lab Immunol.

[55] Wu HC (2014). Correlation of DNA methylation levels in blood and saliva DNA in young girls of the LEGACY Girls study. Epigenetics.

[56] Rozek LS (2014). Epigenetics: relevance and implications for public health. Annu Rev Public Health.

[57] Perera F, Herbstman J (2011). Prenatal environmental exposures, epigenetics, and disease. Reprod Toxicol.

[58] Liu J (2008). Combined inhaled diesel exhaust particles and allergen exposure alter methylation of T helper genes and IgE production in vivo. Toxicol Sci.

[59] Brand S (2012). DNA methylation of TH1/TH2 cytokine genes affects sensitization and progress of experimental asthma. J Allergy Clin Immunol..

[60] Runyon RS (2012). Asthma discordance in twins is linked to epigenetic modifications of T cells. PLoS ONE.

[61] Kohli A (2012). Secondhand smoke in combination with ambient air pollution exposure is associated with increasedx CpG methylation and decreased expression of IFN-gamma in T effector cells and Foxp3 in T regulatory cells in children. Clin Epigenetics.

[62] White GP (2006). CpG methylation patterns in the IFNgamma promoter in naive T cells: variations during Th1 and Th2 differentiation and between atopics and non-atopics. Pediatr Allergy Immunol.

[63] Jones B, Chen J (2006). Inhibition of IFN-gamma transcription by site-specific methylation during T helper cell development. EMBO J.

[64] Lovinsky-Desir S (2014). DNA methylation of the allergy regulatory gene interferon gamma varies by age, sex, and tissue type in asthmatics. Clin Epigenetics.

[65] van den Brink GR (2002). Feed a cold, starve a fever?. Clin Diagn Lab Immunol.

[66] Huang SL, Pan WH (2001). Dietary fats and asthma in teenagers: analyses of the first Nutrition and Health Survey in Taiwan (NAHSIT). Clin Exp Allergy.

[67] Heinrich J (2001). Allergic sensitization and diet: ecological analysis in selected European cities. Eur Respir J.

