# Supplementary material for: Dietary intake is associated with respiratory health outcomes and DNA methylation in children with asthma
Source: Allergy Asthma Clin Immunol. 2017 Feb 27;13:12. doi: 10.1186/s13223-017-0187-8 (PMC5327515; doi:10.1186/s13223-017-0187-8)
Supplement: Supplementary file 1 — Additional file 1: Table S1. Primers and amplification conditions for PCR and pyrosequencing experiments. Figure S1. Description of ARTIS sub-study design. [file 13223_2017_187_MOESM1_ESM.docx]

| Assay | Primers | PCR Conditions |
| --- | --- | --- |
| LINE-1 | F: 5’-TTTGAGTTAGGTGTGGGATATA-3’ | 56°C  44 Cycles |
|  | R: 5’-biotin-AAAATCAAAAAATTCCCTTTC-3’ |  |
|  | S: 5’-AGTTAGGTGTGGGATATAGT-3’ |  |
| IFNγ CpG -186 | F: 5’-biotin-AGATGGTGATAGATAGGTAGGGATGATA-3’ | 55°C  45 Cycles |
|  | R: 5’-TCCCACCAAAATAACACAAATAAACAT-3’ |  |
|  | S: 5’-AAATAAACATAATAAATCTATCTCA-3’ |  |
| IFNγ CpG -54 | F:5’ATGTGTTGTATTTTTTTTGGTTGTTGGTAT-3’ | 55°C  45 Cycles |
|  | R: 5’-biotin-TATCATCCCTACCTATCTATCACCATCTC-3’ |  |
|  | S: 5’ATTGAAGTTTTTTGAGGATT-3’ |  |

Supplement

Table S1. Primers and amplification conditions for PCR and pyrosequencing experiments

Definitions: IFNγ, interferon gamma

Figure S1. Discription of ARTIS sub-study design

A total of 32 subjects representing 32 households were recruited from Western Montana to have 4 in-home visits, 2 per winter, with the introduction of an treatment intervention between winter #1 and #2. In this study we are evaluating the impact of current diet status on asthma health and methylation profiles therefore only data from visits that included the diet survey (i.e. visits B and D) were included in the analysis.
